# Evaluation and development of a novel binocular treatment (I-BiT™) system using video clips and interactive games to improve vision in children with amblyopia (‘lazy eye’): study protocol for a randomised controlled trial

**DOI:** 10.1186/1745-6215-14-145

**Published:** 2013-05-20

**Authors:** Alexander J Foss, Richard M Gregson, Daisy MacKeith, Nicola Herbison, Isabel M Ash, Sue V Cobb, Richard M Eastgate, Trish Hepburn, Anthony Vivian, Diane Moore, Stephen M Haworth

**Affiliations:** 1 Human Factors Research Group, University of Nottingham, University Park, Nottingham NG7 2RD, UK; 2 Nottingham University Hospital NHS Trust, QMC Campus, Derby Road, Nottingham NG7 2RB, UK; 3 Addenbrookes Hospital, Hills Road, Cambridge CB2 0QQ, UK

**Keywords:** Amblyopia, I-BiT™, Randomised clinical trial, Lazy eye, Child, Visual acuity, Binocular

## Abstract

**Background:**

Amblyopia (lazy eye) affects the vision of approximately 2% of all children. Traditional treatment consists of wearing a patch over their ‘good’ eye for a number of hours daily, over several months. This treatment is unpopular and compliance is often low. Therefore results can be poor. A novel binocular treatment which uses 3D technology to present specially developed computer games and video footage (I-BiT™) has been studied in a small group of patients and has shown positive results over a short period of time. The system is therefore now being examined in a randomised clinical trial.

**Methods/design:**

Seventy-five patients aged between 4 and 8 years with a diagnosis of amblyopia will be randomised to one of three treatments with a ratio of 1:1:1 - I-BiT™ game, non-I-BiT™ game, and I-BiT™ DVD. They will be treated for 30 minutes once weekly for 6 weeks. Their visual acuity will be assessed independently at baseline, mid-treatment (week 3), at the end of treatment (week 6) and 4 weeks after completing treatment (week 10). The primary endpoint will be the change in visual acuity from baseline to the end of treatment. Secondary endpoints will be additional visual acuity measures, patient acceptability, compliance and the incidence of adverse events.

**Discussion:**

This is the first randomised controlled trial using the I-BiT™ system. The results will determine if the I-BiT™ system is effective in the treatment of amblyopia and will also determine the optimal treatment for future development.

**Trial registration:**

ClinicalTrials.gov identifier: NCT01702727

## Background

Amblyopia occurs when normal visual experience is disrupted during the critical periods for visual development. It most commonly presents in association with strabismus, anisometropia or both. Several forms of treatment for amblyopia exist but occlusion treatment is the gold standard and involves covering the good eye with a patch for a prescribed period of time each day [1]. The length of patching treatment varies and ranges from 10 minutes daily to all waking hours [2]. The child must wear the patch at home or school and typically, 120 hours of patching treatment results in 1 LogMAR line of improvement in visual acuity [3]. Treatment is prolonged and this contributes to poor compliance. Many children refuse to wear their patch for the prescribed time and this is a major cause of failure of treatment. Many children feel they are stigmatised by the wearing of a patch. Dixon-Woods *et al*. [4] reported patching caused distress and other negative outcomes such as relationship strain. In extreme cases, non-compliance with patching can result in a child having a costly admission to hospital to have supervised patching treatment. In addition, wearing a patch prevents both eyes being used at the same time which eliminates any advantage of binocularity (using the two eyes together as a pair).

Other forms of amblyopia treatment are used such as atropine eye drops and optical penalisation. These are usually chosen as secondary treatments if patching fails, and act by reducing the vision of the good eye in order that the child uses the amblyopic eye to see. All the existing traditional treatments rely on penalisation of the good eye with either a patch, eye drops or through the use of lenses (optical penalisation).

As an alternative to this treatment, we have developed a virtual reality-based system to treat amblyopia where children play special video games and watch DVDs. This interactive binocular treatment system (I-BiT™) uses specially configured software to preferentially stimulate the amblyopic eye without compromising the vision in the good eye. The treatment activity is designed to be enjoyable and, importantly, the child does not have to suffer the unpleasant experience of having their vision limited by the sole use of their amblyopic eye (as they do with patching). The treatment has so far been delivered in the hospital eye clinic but the I-BiT system has the potential to be further developed for use in the home environment in the future.

Previous pilot studies [5] have shown the I-BiT™ system to be highly effective in improving the visual acuity in amblyopic patients. Since we first began our studies, 3D technology has improved and we have recently conducted a pilot study to assess delivery of I-BiT™ treatment using the latest generation of shutter glasses technology [6]. This study showed that all patients who completed their planned treatment (9 of the 10 patients) showed an improvement in visual acuity. These improvements ranged from 0.025 to 0.45 LogMAR units with a mean of 0.18 (sd 0.143) and a median of 0.175.

While the study did have limitations (it was uncontrolled, unblinded and the sample size was small) the results were promising and warranted further investigation in a randomised controlled study. This study will aim to compare the effect of the I-BiT™ game with the I-BiT™ DVD and also to compare the effect of the I-BiT™ game with a control in which the patient plays the game without the amblyopic eye being preferentially stimulated.

## Methods/design

### Objectives of the study

The primary objective of the study is to investigate whether there is any difference in visual acuity in patients treated with I-BiT™ *versus* non-I-BiT™ and also if there is any difference between the two treatment stimuli (interactive game *versus* DVD). Therefore two comparisons will be made: differentiation of the type of I-BiT™ treatment and differentiation of I-BiT™ from non-I-BiT™.

Secondary objectives include examining visual acuity at additional time points (mid-treatment and 4 weeks after treatment has stopped), investigating the proportion of patients showing a response to treatment (a change from baseline of 0.125 or more [7]), the proportion of change in visual acuity, the change in binocular function, the acceptability of the treatment to the patient, compliance with treatment, and the safety profile of the treatment.

### I-BiT™ shutter glasses system

The I-BiT™ system hardware consists of a desktop PC with two monitors, one for the clinician and one for the patient. As with all previous I-BiT™ system prototypes, the clinician monitor is used to control the treatment the patient receives and the patient monitor displays the visual stimuli. The system uses 3D shutter glasses and their corresponding infra-red emitter. The patient monitor is a flat-screen 18-inch 3D monitor with a refresh rate of 120 Hz as required for use with the shutter glasses. The shutter glasses lenses lighten and darken in synchrony with the monitor but faster than the user can perceive. The patient sits on a comfortable gaming beanbag for the duration of their treatment.

This I-BiT™ system relies on the same principle as previous prototypes [8]. Images are presented to both eyes but parts of the image are presented only to the amblyopic eye. The visual scene with the I-BiT™ system is not presented stereoscopically. Instead the 3D technology is used to present a distinct but visually related image to each eye allowing the perception of a dynamic, two-dimensional visual scene. The I-BiT™ system can display video footage and interactive games. A gaming control pad is used for the games. The games have been specifically developed to appeal to children aged 8 years and under. The visual stimuli presented in the current study are described below.

### Video stimulus

The principle employed is that the image is divided into two zones. There is an outer ‘border’ termed a locking stimulus which is presented to both eyes while the inner part of the screen presents the video footage to the amblyopic eye. Images within the border can be selectively shown to either eye to act as a control to ensure binocular viewing. The DVD transparency settings for the non-amblyopic eye can be adjusted for those with dense suppression if required. This is to encourage continued viewing and fixation with the amblyopic eye. To increase compliance the I-BiT system has a built in DVD player and this allows children to watch a DVD of their own choice.

### Game stimulus

An interactive game called ‘Nux’ will be used to provide the game-play. In Nux, a player moves through a colourful two-dimensional space-like environment. Points are rewarded for collecting coins and shooting enemies and deducted for colliding with enemies and obstacles (for example, asteroids). Through the I-BiT™ system, the player and the background are shown to both eyes but the obstacles, enemies and coins are shown only to the amblyopic eye. Therefore, in order for the child to play the game successfully, they must use their amblyopic eye. If the patient is unable to play due to dense suppression or severely reduced visual acuity in their amblyopic eye then the settings can be adjusted so that a proportion of the objects (coins and so on.) are seen by the non-amblyopic eye. In the non-I-BiT game version (control arm) both eyes receive identical stimulation.

### Design

The study is a randomised parallel group, double-masked design. Eligible patients are randomised to one of three treatments:

• I-BiT™ game

• Non-I-BiT™ game

• I-BiT™ DVD

**Figure 1:**
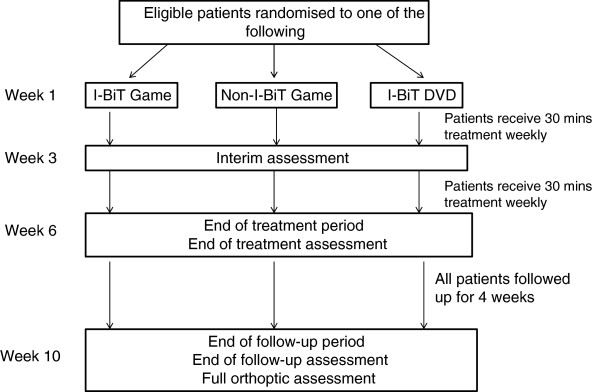
Outline of study procedures.

They will receive their randomised treatment weekly for 6 weeks, for a 30-minute period (Figure 1). Their visual acuity will be assessed pre-treatment, after three treatments, after six treatments and 4 weeks after their final treatment. Visual acuity will be assessed by an independent assessor who has no knowledge of the randomised treatment. The orthoptist treating the patient will have no knowledge of the results of the patient’s visual acuity assessments. The study is therefore masked.

Two comparisons are to be made: I-BiT™ game *vs.* non-I-BiT™ game and I-BiT™ game *vs.* I-BiT™ DVD.

### Eligibility criteria

#### Inclusion criteria

• Diagnosis of anisometropic, strabismic or mixed amblyopia as made by an orthoptist (difference of 2 LogMAR units or more using a LogMAR crowded test)

• Male or female

• Aged 4 to 8 years inclusive

• Participant’s parent or guardian is willing and able to give informed consent for participation in the study

### Exclusion criteria

The participant may not enter the study if any of the following apply:

• Stimulus deprivation amblyopia

• Organic lesions of the eye preventing the establishment of good vision (for example, media opacities, abnormalities in the fundus or optic nerve)

• Lesions of the brain preventing the establishment of good vision (for example, cortical visual impairment)

• Patients diagnosed with photosensitive epilepsy

• Patients diagnosed with or suspected of having conjunctivitis

• Loss of suppression at filter 4 or less as measured with the Sbisa bar

• Establishment of normal vision by refractive adaptation (wearing glasses after presentation)

• Inability to comply with the follow-up visits required

• Refusal to accept randomisation

• Have participated in a previous study examining I-BiT™ treatment

### Study procedures

#### Screening and eligibility assessments

Potential participants will be identified by various methods including identification by members of the clinical team or by contacts made through the website and via posters in clinic. Informed consent will be obtained and eligibility assessed.

### Baseline assessments

A full orthoptic assessment a maximum of 1 month prior to the start of the study must be available/performed. This assessment must include:

• Uniocular assessment of visual acuity with a LogMAR test (aided with glasses if applicable)

• Cover test (with and without glasses if applicable)

• Ocular motility

• Assessment of binocular functions

• Visuscope assessment

• Sbisa bar (if applicable)

### Randomisation and codebreaking

Subject numbers will be assigned sequentially as each subject enters the study. The subjects will be assigned to a study treatment arm through an independently-developed web-based randomisation system. Randomisation will be performed after eligibility has been checked prior to first treatment, and will be equal and stratified according to whether the patient has had previous treatment for their amblyopia. The research orthoptist will be the only member of the study team who will have knowledge of the randomised treatment.

### Subsequent assessments

Patients will receive their randomised treatment for 30 min each week for 6 weeks. In addition to their baseline assessment, they will have assessments at the following visits, all performed by an independent assessor.

### Week 1 (pre-treatment)

Uniocular assessment of visual acuity with a LogMAR test (aided with glasses if applicable) prior to first treatment.

### Week 3 (mid-treatment)

Uniocular assessment of visual acuity with a LogMAR test (aided with glasses if applicable) after third treatment.

If at this visit, visual acuity in the amblyopic eye has regressed by 0.1 LogMAR units or more, then the participants should be withdrawn from the study.

### Week 6 (end of treatment)

Uniocular assessment of visual acuity with a LogMAR test (aided with glasses if applicable) after sixth treatment.

### Week 10 (follow-up)

Full orthoptic assessment.

The independent assessor may perform additional tests such as the Sbisa bar and Frisby test if clinically indicated. The research orthoptist will record details of the treatment at each visit and ascertain whether the patient has had any adverse events.

At week 10 (follow-up), participation in the study will be complete for the patient and they will be returned to the care of the orthoptic department. Patients with residual amblyopia will receive standard treatment in line with departmental guidelines.

The patient may be withdrawn from the study at any point at the clinician’s discretion.

### Outcomes

#### Primary outcome

The primary variable is visual acuity. Visual acuity is measured in units of 0.025 (which equates to one letter). Improvement in visual acuity is measured as an increase of 0.025 LogMAR units.

The primary endpoint is the change from baseline to the end of treatment (6 weeks) in visual acuity.

The secondary endpoints are:

• Change in visual acuity from baseline to the end of the follow-up period (week 10)

• Change in visual acuity from baseline to week 3

• Proportion of patients showing a clinically important change in visual acuity (a change of 0.125 LogMAR units or more) at weeks 3, 6 and 10

• Proportion of change in visual acuity [9] at weeks 3, 6 and 10

• Change in binocular functions from baseline to week 6/week 10

• Proportion of patients reporting outcomes in each item of the patient satisfaction questionnaire

• Percentage compliance with treatment

• Proportion and type of adverse event

Proportion of change is measured as follows:

$$\mathrm{Proportion}\;\mathrm{of}\;\mathrm{change}=VA_{\mathrm{as}}-VA_{\mathrm{ae}}/VA_{\mathrm{as}}-VA_{\mathrm{fe}}$$

VA_as_ is vision of amblyopic eye at baseline, VA_ae_ is vision of amblyopic eye at the ‘end of treatment’ and VA_fe_ is the vision of the non-amblyopic eye at the’ end of treatment’ [9]. ‘End of treatment’ is the time point the change is being measured up to, that is, weeks 3, 6 and 10.

### Safety assessment

**Figure 2:**
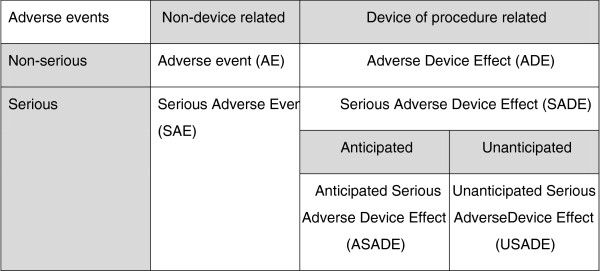
Classification of adverse effects.

All adverse events will be recorded. They will be categorised according to whether they are device related according to the Figure 2.

Events which could be considered to be anticipated due to the hypothesised mechanism of action of the treatment include nausea, photosensitive epilepsy, headache, diplopia and reverse amblyopia. None of these events have been observed in previous studies.

### Sample size

Assuming a standard deviation (SD) of 0.25 [8], 25 patients per arm would be required to detect a minimum difference of 0.2 LogMAR units at the 5% significance level (two-sided) with 80% power. Therefore a total of 75 patients would be required.

### Statistical methods

The primary endpoint is considered confirmatory with secondary endpoints considered supportive.

The primary analyses will be performed on the intention-to-treat (ITT) population. Supportive analyses may be performed on a per protocol (PP) population defined prior to code-break.

Except where a patient has been withdrawn from the study or otherwise documented, all missing data will be assumed to be missing completely at random (MCAR). The primary analyses will not use any imputation methods to compensate for missing data.

There will be two primary comparisons: I-BiT™ games *vs.* non-I-BiT™ games and I-BiT™ DVD *vs.* I-BiT™ games. Both these analyses will comprise ANCOVA using baseline visual acuity as a co-variate, where distributional assumptions are met. If these assumptions are not met, a suitable transformation will be applied or a non-parametric method used. There will be no correction for multiplicity.

Summary statistics including mean, median, SD and 95% confidence intervals will be provided, in addition to appropriate graphical displays.

All datasets will be defined in a blinded manner prior to database lock.

Further details of the statistical analyses will be provided in the statistical analysis plan which will be finalised prior to database lock.

### Monitoring

No data monitoring committee will be appointed for this study due to the expected low incidence of adverse events. Routine data monitoring will be performed in accordance with the sponsor’s standard operating procedures.

### Ethical considerations

The study has received approval from both the Research Ethics Committee and Medicines and Health Regulatory Authority (MHRA) and will be conducted in accordance with the Declaration of Helsinki, ICH GCP and ISO14155.

## Discussion

This is the first randomised controlled study using the I-BiT™ system. It aims to determine whether the improvements in visual acuity observed in previous, uncontrolled studies of I-BiT™ technology are maintained under strict clinical trial conditions. It also aims to determine whether there is a difference between playing games and watching DVDs using I-BiT™ technology, and whether there is a difference between playing games using and not using the I-BiT™ technology and therefore identify which is the optimal treatment to develop.

Compliance is a huge issue with conventional patching treatment which impacts on the efficacy of the treatment. Therefore this study also aims to determine the compliance of patients undergoing each of the treatments.

The study also aims to determine whether there are any adverse effects which could be related to I-BiT™. It has been hypothesised that there is a theoretical risk of adverse events such as nausea, photosensitive epilepsy, headache, diplopia and reverse amblyopia. However, none of these events have been observed in previous studies.

It has not been possible to fully blind the study. However, the masking procedures ensure that clinical assessments are made in a blinded manner and therefore should not be subject to bias. The treating orthoptist will have no access to assessment results either through the database or through medical notes and indeed controls are in place to ensure that the treating orthoptist cannot treat these patients after they complete the study and thereby gain access to their resultant visual acuity measurement.

This study looks specifically at patients between the ages of 4 and 8 years. Cleary *et al.*[10] found improvements in older children in a pilot study with an I-BiT™ prototype system. Therefore it is possible that improvements could be gained in patients older than 8 years with the current I-BiT™ system. However, there are no plans formally assess this at present, the priority being to develop an optimal treatment regimen for children.

The results of this study will inform further development. It will be necessary to determine an optimal treatment regimen which may involve more frequent visits or a larger number of treatments in order to provide a long-term effect. It will also be necessary to follow-up patients in the longer term to ensure maintenance of beneficial effects.

If I-BiT™ is shown to be an effective treatment for amblyopia there is great potential for developing this treatment in a number of settings. While making available in hospital orthoptic clinics, there is also the potential that it could be used in high street opticians throughout Europe and even the potential to develop a home-based treatment regimen. There is therefore the opportunity to make improvements in both vision and quality of life for children, but to also decrease treatment costs.

## Trial status

As of 6 November 2012, 21 patients have been randomised. Recruitment will continue until 75 patients are randomised.

## Abbreviations

ADE: Adverse device effect; AE: Adverse event; ANCOVA: Analysis of covariance; ASADE: Anticipated Serious Adverse Device effect; I-BiT™: Interactive binocular treatment system; ICH GCP: International Conference on Harmonisation Good Clinical Practice; ITT: Intention to treat; LOCF: Last observation carried forwards; MCAR: Missing completely at random; MHRA: Medicines and Health Regulatory Authority; PP: Per protocol; SADE: Serious adverse device effect; SAE: Serious adverse event; SD: Standard deviation; USADE: Unanticipated serious adverse device effect; VA: Visual acuity.

## Competing interests

The patent for the I-BiT™ system is held jointly by Nottingham University Hospitals NHS Trust and the University of Nottingham which employ AF, DMacK, NH, IA, SC, RE and TH.

## Authors’ contributions

RG and NH conceived the study design which was further developed by TH, DMacK and IA. All the authors made a substantial contribution to the clinical information plan development. All authors read and approved the final manuscript.

## Authors’ information

Stephen Haworth smhaworth@ntlworld.com - Retired Ophthalmologist.
